# Finding modifiable predictors of treatment dropout: the role of the therapeutic alliance, readiness to change and acceptance of treatment approach

**DOI:** 10.1186/2050-2974-3-S1-O37

**Published:** 2015-11-23

**Authors:** Louise Pannekoek, Sue Byrne, Anthea Fursland

**Affiliations:** 1 University of Western Australia, Australia; 2 Centre for Clinical Interventions, Austrlia

## 

Although abundant, research has been unable to identify consistent predictors of dropout from eating disorder treatment. One potential reason for this is the bias towards research into fixed, individual, patient factors (e.g. personality traits and duration of illness). This quantitative study aimed to confirm results of a qualitative study, which suggested that three process factors (therapeutic alliance, readiness to change and acceptance of treatment approach) were important contributors to patients' decision to drop out of treatment. The study involved data from 332 consecutive referrals (98% female) to a public outpatient eating disorder service, who received individual Cognitive Behavioural Therapy between 2005 and 2014. Almost 40% of the sample dropped out of treatment (defined as non-mutual termination). Binary logistic regressions showed that patient ratings on items related to the therapeutic alliance and acceptance of treatment approach (as measured by the Helping Alliance Questionnaire, Credibility / Expectancy Questionnaire, and a measure of the patient's perception of therapist) were significantly associated with treatment dropout. However, readiness to change items, and total questionnaire scores, were not significant predictors. Issues in the measurement and self-report of all of these factors may mask the true relationship between process factors and treatment dropout.

